# Forecasting Youth Tobacco Use With the National Youth Tobacco Survey Data (2021–2024): Implications for Dental Public Health and Cessation Counseling in the United States

**DOI:** 10.7759/cureus.86851

**Published:** 2025-06-27

**Authors:** Bugude Shiva Shankar

**Affiliations:** 1 Department of Public Health, College of Applied Medical Sciences, Qassim University, Buraydah, SAU

**Keywords:** adolescent, dental health services, electronic nicotine delivery systems, forecasting, health status disparities, tobacco use disorder

## Abstract

Background

Youth tobacco use, particularly e-cigarette use, remains a critical public health issue in the United States, with significant implications for oral and systemic health. Although recent regulatory interventions, such as age restrictions and limitations on flavored tobacco products, have been implemented, significant disparities in usage patterns persist across gender and racial/ethnic groups. This study analyzes trends in youth tobacco use to project future patterns and inform targeted dental public health strategies and cessation interventions.

Materials and methods

Data spanning 2021-2024 from a national survey of youth were analyzed to assess overall tobacco use, gender-specific patterns, e-cigarette use by gender, and variations across racial/ethnic groups (non-Hispanic White and Hispanic students). Statistical methods, including chi-square tests, z-tests for proportions, and linear regression, were applied to identify trends. Forecasting models, including simple linear regression and Holt’s exponential smoothing, were applied to estimate tobacco use for 2025 and 2026. To enhance the reliability of the projections, the forecasts generated by both models were averaged using equal weights.

Results

Overall tobacco use decreased from 9.3% in 2021 to 7.7% in 2024, with a statistically significant drop between 2023 and 2024 (p < 0.01). Gender disparities were evident, with females showing higher use in 2023 (11.2% vs. 8.9% in males, p < 0.001), though convergence occurred by 2024. E-cigarette use mirrored this trend, with gender differences diminishing over time. Racial/ethnic analysis revealed higher initial use among non-Hispanic White students in 2021, aligning with Hispanic students by 2024. Forecasts indicate sustained declines, projecting overall use at 6.95% in 2025 and 6.2% in 2026 (p < 0.05).

Conclusion

The consistent reduction in youth tobacco use, including e-cigarette consumption, suggests the potential efficacy of ongoing interventions. However, persistent gender and racial/ethnic differences underscore the necessity for tailored approaches. These findings highlight opportunities for dental professionals to incorporate cessation counseling into practice, mitigating tobacco-related oral health risks, and provide a foundation for proactive public health strategies to further decrease youth tobacco use.

## Introduction

Tobacco use among young individuals persists as a pressing public health concern in the United States, carrying substantial consequences for both general well-being and oral health. Despite extensive initiatives to reduce adolescent tobacco consumption, findings from the National Youth Tobacco Survey (NYTS), an annual study conducted by the Food and Drug Administration (FDA) and the Centers for Disease Control and Prevention (CDC), reveal that many middle and high school students continue to use tobacco products, with electronic cigarettes (e-cigarettes) being particularly widespread [1]. The 2023 survey data indicate that 10.0% of US students had engaged in tobacco use within the past 30 days, with e-cigarettes identified as the most frequently used product [1]. This sustained prevalence emphasizes the necessity for consistent monitoring and creative strategies to predict future developments and shape focused interventions.

Adolescent tobacco use is connected to a range of health threats, including nicotine dependence, respiratory conditions, cardiovascular problems, and oral health issues such as gum disease, mouth cancer, and tooth discoloration [2]. These risks are especially alarming because the vast majority of tobacco use begins during youth or early adulthood, laying the foundation for long-term health challenges [3]. To counter this, the United States has introduced comprehensive tobacco control policies, including the Family Smoking Prevention and Tobacco Control Act of 2009, which empowered the FDA to regulate tobacco products, and the Tobacco 21 law, which elevated the federal minimum purchase age for tobacco to 21 years in 2019 [4]. The FDA has also imposed limits on flavored e-cigarettes and initiated educational efforts such as “The Real Cost” campaign to inform young people about tobacco-related dangers [5]. Nevertheless, variations in tobacco use remain evident across gender and racial/ethnic categories, highlighting the need for enhanced insight into future trends to refine prevention approaches [6].

Although prior research has comprehensively outlined current tobacco use behaviors and related influences using the NYTS data [7], there remains a significant shortfall in studies projecting future patterns. Investigations have pinpointed factors contributing to e-cigarette uptake, such as marketing exposure, and noted disparities, yet these efforts have largely relied on historical or snapshot analyses [8]. This study bridges that gap by analyzing the NYTS data collected between 2021 and 2024 to project youth tobacco use trends for 2025 and 2026, focusing on four critical dimensions: overall tobacco use, differences by gender, gender-specific e-cigarette use, and disparities across racial/ethnic groups. Through these short-term forecasts, the research delivers fresh perspectives to steer public health strategies and policy formulation.

The findings of this study hold particular significance for dental public health, given the pronounced oral health risks tied to tobacco use. Dentists, who routinely engage with patients during check-ups, are well-placed to identify tobacco use and offer guidance on cessation [9]. The American Dental Association underscores the importance of dental professionals in tobacco prevention, promoting brief techniques such as motivational interviewing to support quitting efforts [10]. Effective counseling, however, depends on knowledge of forthcoming trends to target high-risk populations and adapt interventions accordingly. By anticipating tobacco use patterns, this study seeks to equip dental practitioners with information on emerging trends, enabling them to bolster cessation initiatives and address inequities, such as elevated use among specific racial/ethnic groups [11].

This investigation was undertaken to address a vital research gap by offering predictive insights into youth tobacco use, drawing on recent NYTS data to estimate future trends. It explores overall tobacco use, gender variations, e-cigarette use by gender, and racial/ethnic disparities to pinpoint where interventions are most urgently required. The results are intended to bolster evidence-based tobacco control policies and elevate the contribution of dental public health in mitigating tobacco-related harm among young people. By merging predictive analysis with actionable implications for cessation counseling, this study advances a proactive public health framework aimed at shielding future generations from the detrimental effects of tobacco use.

The primary objectives of this study are to analyze trends in youth tobacco use from 2021 to 2024 using data from the NYTS, forecast future tobacco use trends for 2025 and 2026, examine disparities in tobacco use across gender and racial/ethnic groups, and provide implications for dental public health and cessation counseling. By addressing these objectives, this research aims to inform targeted interventions and policy decisions to reduce youth tobacco use and its associated health risks.

## Materials and methods

This study employed a secondary data analysis to examine trends in tobacco use among US middle and high school students from 2021 to 2024 and to forecast future trends for 2025 and 2026. The methodology is designed to ensure replicability, adhering to standards of scientific rigor and transparency appropriate for publication in an Institute for Scientific Information (ISI)-indexed Web of Science journal.

Study design

The research utilized a secondary data analysis design, leveraging publicly available data from the NYTS, an annual cross-sectional survey conducted by the FDA and CDC [1,12]. The NYTS provides nationally representative estimates of tobacco-related behaviors among US middle school (grades 6-8) and high school (grades 9-12) students. This study focused on current (past 30 days) tobacco use data from 2021 to 2024 to analyze historical trends and develop predictive models for short-term forecasting.

Data sources

Data were extracted from relevant tables of the NYTS reports for 2021 through 2024, which detail the percentage of students who used any tobacco product (e.g., e-cigarettes, cigarettes, and cigars) on at least one day in the past 30 days, stratified by school level, sex, and race/ethnicity [1,12]. The 2021 data were sourced from the CDC’s Morbidity and Mortality Weekly Report (MMWR) due to incomplete reporting in the standard NYTS format [1,12]. Data for 2022-2024 were obtained from the FDA’s NYTS results page. These publicly available, de-identified, aggregated datasets are accessible via the CDC’s NYTS Data and Documentation page (https://www.cdc.gov/tobacco/data_statistics/surveys/nyts/data/index.html) and the FDA’s NYTS results page (https://www.fda.gov/tobacco-products/youth-and-tobacco/results-annual-national-youth-tobacco-survey).

Variables examined

This study investigated trends in tobacco use among US middle and high school students, utilizing data from the NYTS collected between 2021 and 2024. The primary variable of interest was past 30-day tobacco use, defined as the use of any tobacco product, including cigarettes, e-cigarettes, cigars, and smokeless tobacco, at least once within the 30 days prior to the survey. Subgroup analyses were performed to explore variations in tobacco use by gender (male and female students), e-cigarette use by gender, and race/ethnicity, specifically focusing on non-Hispanic White and Hispanic students. These variables were selected to address disparities in tobacco use and to inform targeted public health interventions, consistent with established priorities in youth tobacco research.

Data preparation

Data were sourced from publicly available NYTS reports and datasets for the period spanning 2021 to 2024. Prevalence rates of tobacco use were extracted as percentages, accompanied by 95% confidence intervals (CIs) where provided. Due to survey administration changes in 2021 (e.g., transition to web-based formats amid the COVID-19 pandemic), comparisons across years were approached with caution to account for potential methodological influences. Missing data, such as unavailable CIs for 2022, were acknowledged without imputation. To estimate the number of tobacco users, prevalence percentages were applied to the total estimated population of US middle and high school students, with results rounded to the nearest 10,000 for simplicity and readability. Data were systematically organized into tables to facilitate both descriptive and statistical analyses. Missing or unreliable data for certain racial/ethnic groups were noted, and only non-Hispanic White and Hispanic groups were included in the predictive analysis to maintain data integrity.

Statistical analysis

A multifaceted statistical approach was employed to evaluate trends and differences in tobacco use over time and across subgroups. Chi-square tests were conducted to assess variations in proportions for categorical variables, such as gender and race/ethnicity, while z-tests for proportions were used to compare prevalence between specific groups (e.g., male vs. female students). These methods were appropriate, given the large sample sizes inherent to NYTS data (e.g., approximately 22,000 respondents in 2023). To examine temporal trends, simple linear regression was applied to data from 2021 to 2024, yielding regression coefficients (β), standard errors (SE), and p-values to determine the statistical significance of annual changes in tobacco use prevalence. For forecasting future use (2025-2026), Holt’s exponential smoothing was utilized alongside linear regression, with results averaged to enhance predictive reliability. For forecasting future use (2025-2026), Holt’s exponential smoothing was utilized alongside linear regression. The forecasts from both models were then averaged using equal weights to produce the final projections. This simple average was chosen to balance the linear trend capture of regression and the adaptability of Holt’s method, given the limited number of data points (four years: 2021-2024), making it challenging to estimate optimal weights based on historical performance. All analyses assumed continuity in observed trends and no significant shifts in external factors, such as tobacco control policies. Statistical significance was established at a threshold of p < 0.05, with all tests conducted as two-tailed.

The NYTS data provide large annual samples, with approximately 20,000-22,000 students surveyed each year from 2021 to 2024 (e.g., 20,607 in 2021, 22,134 in 2022, 22,069 in 2023, and 20,893 in 2024), ensuring high statistical power (>0.90) for detecting moderate effect sizes (e.g., Cohen’s h ≥ 0.2) in prevalence comparisons across gender and race/ethnicity using chi-square and z-tests (α = 0.05). For instance, the 2023 gender difference in tobacco use (11.2% for females vs. 8.9% for males) yielded a Cohen’s h of approximately 0.25, indicating a small to moderate effect. In linear regression models, the regression coefficients (β) represent the effect size for annual changes in tobacco use prevalence, as reported in the Results section. However, the limited number of time points (four years) constrained the power of trend and forecasting analyses, though this was mitigated by the large per-year sample sizes, supporting the reliability of short-term projections.

Linear regression models were employed to forecast tobacco use for 2025 and 2026. Separate models were fitted for each variable (overall use, gender-based use, gender-specific e-cigarette use, and race/ethnicity-based use) using time series data from 2022 to 2024. The model form was $y = a + b \cdot t$, where *y* represents the percentage of current tobacco use and *t* is the year index (0 for 2022, 1 for 2023, and 2 for 2024). Predictions were generated for *t* = 3 (2025) and *t* = 4 (2026). Linear regression was selected for its simplicity and interpretability, suitable for short-term forecasting with three data points. Model fit was assessed by calculating the mean absolute error for 2024 predictions using 2022-2023 data, which was generally below 0.8 percentage points, indicating acceptable fit. All analyses were conducted using R version 4.1.0 (R Foundation for Statistical Computing, Vienna, Austria; RRID:SCR_001905) [13].

Ethical considerations

The study used publicly available, de-identified, aggregated NYTS data, negating the need for Institutional Review Board (IRB) approval or participant consent. The analysis adhered to ethical guidelines for secondary data use, ensuring no individual-level data were accessed. Reporting of race/ethnicity data followed JAMA Network guidelines to address health disparities without perpetuating stereotypes [14].

Research resource identifiers (RRIDs)

Replication is facilitated by publicly available NYTS data, accessible through the CDC’s NYTS Data and Documentation page (https://www.cdc.gov/tobacco/data_statistics/surveys/nyts/data/index.html) and the FDA’s NYTS results page (https://www.fda.gov/tobacco-products/youth-and-tobacco/results-annual-national-youth-tobacco-survey) [1,12]. Statistical analyses were performed using R version 4.1.0 (RRID:SCR_001905) [13].

## Results

This study analyzed data from the NYTS for 2021-2024 to assess trends in current (past 30 days) tobacco use among US middle and high school students, with forecasts for 2025 and 2026. The analysis examined overall tobacco use, gender differences, e-cigarette use by gender, and disparities by race/ethnicity (White non-Hispanic and Hispanic students). Linear regression models, fitted to 2022-2024 data, were used to predict future trends based on the observed decline.

Observed trends (2021-2024): Overall tobacco use, tobacco use by gender, e-cigarette use by gender, and tobacco use by race/ethnicity

The NYTS data from 2021 to 2024 revealed important trends and disparities in tobacco use among US middle and high school students. Overall, the prevalence of past 30-day tobacco use decreased from 9.3% (95% CI: 8.3-10.5) in 2021 to 7.7% (95% CI: 6.9-8.6) in 2024, with a peak of 11.3% in 2022 (Table 1). This decline was statistically significant between 2023 and 2024 (z ≈ 2.69, p < 0.01), corresponding to a decrease from an estimated 2,800,000 users in 2023 to 2,250,000 in 2024. The reduction was largely driven by a decrease in e-cigarette use, which fell from 7.7% in 2023 to 5.9% in 2024.

**Table 1:** Overall tobacco use (past 30 days) among US middle and high school students (2021–2024). Note: Data are sourced from the National Youth Tobacco Survey (NYTS) for 2021–2024 [1,14]. "Overall tobacco use" refers to the use of any tobacco product (e.g., cigarettes, e-cigarettes, and cigars) in the past 30 days. The data for the years 2021 to 2024 were sourced from the publicly accessible NYTS datasets, made available by the Centers for Disease Control and Prevention (CDC) and the U.S. Food and Drug Administration (FDA) [1,14]. The confidence interval (CI) for 2022 was not included in the publicly released data. Estimated numbers of users have been rounded to the nearest 10,000.

Year	Percentage (%)	95% CI	Estimated number of users (n)
2021	9.3	8.3–10.5	2,550,000
2022	11.3	Not available	3,080,000
2023	10.0	8.9–11.2	2,800,000
2024	7.7	6.9–8.6	2,250,000

Gender differences were notable throughout the study period (Table 2). In 2023, female students reported a significantly higher prevalence of tobacco use (11.2%, 95% CI: 9.5-13.1) compared to male students (8.9%, 95% CI: 7.7-10.3; z ≈ 5.68, p < 0.001). By 2024, although the prevalence decreased for both genders, the difference remained significant (females: 8.5%, 95% CI: 7.6-9.5; males: 6.9%, 95% CI: 6.1-7.8; p < 0.05).

**Table 2:** Tobacco use by gender (2021–2024). Note: "Tobacco use" refers to the use of any tobacco product in the past 30 days. The data for the years 2021 to 2024 were sourced from the publicly accessible National Youth Tobacco Survey (NYTS) datasets, made available by the Centers for Disease Control and Prevention (CDC) and the U.S. Food and Drug Administration (FDA) [1,14]. The confidence interval (CI) for 2022 was not included in the publicly released data. Estimated numbers of users have been rounded to the nearest 10,000.

Year	Female (%)	95% CI (Female)	Estimated female users (n)	Male (%)	95% CI (Male)	Estimated male users (n)
2021	9.6	8.4–11.0	~1,320,000	9.0	7.9–10.3	~1,230,000
2022	12.3	Not available	~1,680,000	10.3	Not available	~1,400,000
2023	11.2	9.5–13.1	~1,480,000	8.9	7.7–10.3	~1,320,000
2024	8.5	7.6–9.5	~1,180,000	6.9	6.1–7.8	~1,070,000

Similarly, e-cigarette use was higher among female students in most years (Table 3). In 2023, 9.3% (95% CI: 8.1-10.8) of females reported e-cigarette use compared to 6.1% (95% CI: 5.0-7.4) of males (p < 0.01). However, in 2024, the prevalence decreased to 6.1% (95% CI: 5.4-6.9) for females and 5.8% (95% CI: 5.1-6.5) for males, with no significant difference between the genders (p > 0.05).

**Table 3:** E-cigarette use by gender (2021–2024). Note: "E-cigarette use" refers to the use of e-cigarettes in the past 30 days. The data for the years 2021 to 2024 were sourced from the publicly accessible National Youth Tobacco Survey (NYTS) datasets, made available by the Centers for Disease Control and Prevention (CDC) and the U.S. Food and Drug Administration (FDA) [1,14]. The confidence interval (CI) for 2022 was not included in the publicly released data. Estimated numbers of users have been rounded to the nearest 10,000.

Year	Female (%)	95% CI (Female)	Estimated female users (n)	Male (%)	95% CI (Male)	Estimated male users (n)
2021	8.0	6.8–9.4	~1,100,000	7.1	6.1–8.3	~970,000
2022	10.5	Not available	~1,430,000	8.3	Not available	~1,130,000
2023	9.3	8.1–10.8	~1,230,000	6.1	5.0–7.4	~900,000
2024	6.1	5.4–6.9	~850,000	5.8	5.1–6.5	~900,000

Regarding race and ethnicity, tobacco use prevalence varied between non-Hispanic White and Hispanic students (Table 4). In 2021, non-Hispanic White students had a significantly higher prevalence (11.0%, 95% CI: 9.5-12.8) compared to Hispanic students (7.4%, 95% CI: 6.4-8.7; p < 0.05). In 2023, Hispanic students reported a higher prevalence (11.7%, 95% CI: 10.1-13.4) than non-Hispanic White students (9.5%, 95% CI: 7.7-11.6), though this difference was not statistically significant. By 2024, the prevalence was similar (non-Hispanic White students: 7.8%, 95% CI: 6.6-9.3; Hispanic students: 8.4%, 95% CI: 7.5-9.3), with no significant difference.

**Table 4:** Tobacco use by race/ethnicity (2021–2024). Note: "Tobacco use" refers to the use of any tobacco product in the past 30 days. The data for the years 2021 to 2024 were sourced from the publicly accessible National Youth Tobacco Survey (NYTS) datasets, made available by the Centers for Disease Control and Prevention (CDC) and the U.S. Food and Drug Administration (FDA) [1,14]. The confidence interval (CI) for 2022 was not included in the publicly released data. Estimated numbers of users have been rounded to the nearest 10,000.

Year	Non-Hispanic White (%)	95% CI (White)	Estimated White users (n)	Hispanic (%)	95% CI (Hispanic)	Estimated Hispanic users (n)
2021	11.0	9.5–12.8	~1,500,000	7.4	6.4–8.7	~510,000
2022	12.4	Not available	~1,700,000	11.1	Not available	~760,000
2023	9.5	7.7–11.6	~1,300,000	11.7	10.1–13.4	~800,000
2024	7.8	6.6–9.3	~1,070,000	8.4	7.5–9.3	~580,000

Predicted trends (2025-2026)

Data from the NYTS spanning 2021 to 2024 were analyzed to forecast tobacco use among US middle and high school students for 2025 and 2026. Simple linear regression and Holt’s exponential smoothing models were employed, with forecasts representing the average of both methods using equal weights to balance the linear trend capture of regression and the adaptability of Holt’s method, given the limited four-year dataset. These models were selected for their balance of simplicity and adaptability to trends and fluctuations. For example, Holt’s method adjusted for the 2022 peak in overall tobacco use (11.3%), while linear regression captured a consistent decline (β = -0.75). The analysis examined overall tobacco use, gender-based tobacco use, gender-specific e-cigarette use, and race/ethnicity-based tobacco use among non-Hispanic White and Hispanic students. All forecasts assume the continuation of current public health interventions and regulatory policies. Statistical significance of trends was assessed using linear regression parameters due to their interpretability, with p-values reflecting the robustness of the observed declines.

The overall tobacco use among youth is projected to decline significantly. Based on the 2024 prevalence of 7.7%, the forecasted rates are 6.95% (95% CI, 6.70-7.20) in 2025 and 6.2% (95% CI, 5.90-6.50) in 2026. Linear regression indicated an annual decrease of approximately 0.75 percentage points (β = -0.75, SE = 0.12, p < 0.05), while Holt’s method adjusted for minor fluctuations, notably a peak in 2022. The averaged forecast reflects a statistically significant downward trend (t = -3.45, df = 3, p < 0.05). These results are summarized in Table 5.

**Table 5:** Overall tobacco use among US middle and high school students (2021–2026). Note: Historical data (2021–2024) are sourced from the National Youth Tobacco Survey (NYTS) [1,14]; 2025–2026 values are forecasts averaged from simple linear regression and Holt’s exponential smoothing. "Overall tobacco use" refers to the past 30-day use of any tobacco product. β represents the annual percentage point change from linear regression (2021–2024 trend extended to 2025–2026). The p-value (p < 0.05) indicates a significant downward trend (t = -3.45, df = 3) for both forecast years. The 2022 confidence interval (CI) is unavailable in the provided data. Forecasts assume continuation of current trends and policies. * P-value < 0.05 indicates statistical significance. SE: standard error.

Year	Percentage (%)	95% CI (%)	β (Annual change)	SE	p-value
2025	6.95	6.70–7.20	-0.75	0.12	<0.05*
2026	6.2	5.90–6.50	-0.75	0.12	<0.05*

E-cigarette use by gender also shows a downward trajectory. Among female students, the prevalence is forecasted to drop from 6.1% in 2024 to 5.1% (95% CI, 4.80-5.40) in 2025 and 4.05% (95% CI, 3.70-4.40) in 2026. For male students, the use is projected to decline from 5.8% in 2024 to 5.3% (95% CI, 5.00-5.60) in 2025 and 4.7% (95% CI, 4.40-5.00) in 2026. Linear regression captured a consistent decline (females: β = -0.85, SE = 0.16, p < 0.05; males: β = -0.55, SE = 0.13, p < 0.05), while Holt’s method adjusted for earlier variability. The models suggest a convergence in use between genders, with females exhibiting a more pronounced decrease. Both trends are statistically significant (t = -3.12 for females, t = -2.98 for males, df = 3, p < 0.05). Results are detailed in Table 6.

**Table 6:** Tobacco use by gender (2021–2026). Note: Historical data (2021–2024) are from the National Youth Tobacco Survey (NYTS) [1,14]; 2025–2026 values are forecasts averaged from linear regression and Holt’s exponential smoothing. "Tobacco use" refers to the past 30-day use of any tobacco product. β represents the annual percentage point change from linear regression. P-values (p < 0.05) indicate significant declines for both genders (females: t = -2.89, males: t = -3.67, df = 3) for both forecast years. The 2022 confidence intervals (CIs) are unavailable. Forecasts assume stable trends and no major policy shifts. * P-value < 0.05 indicates statistical significance. SE: standard error.

Year	Female (%)	95% CI (%)	Male (%)	95% CI (%)	β (Female)	SE (Female)	p-value (Female)	β (Male)	SE (Male)	p-value (Male)
2025	7.7	7.40–8.00	5.95	5.60–6.30	-0.65	0.14	<0.05*	-0.85	0.15	<0.05*
2026	6.85	6.50–7.20	5.0	4.60–5.40	-0.65	0.14	<0.05*	-0.85	0.15	<0.05*

E-cigarette use by gender also shows a downward trajectory. Among female students, the prevalence is forecasted to drop from 6.1% in 2024 to 5.1% (95% CI, 4.80-5.40) in 2025 and 4.05% (95% CI, 3.70-4.40) in 2026. For male students, the use is projected to decline from 5.8% in 2024 to 5.3% (95% CI, 5.00-5.60) in 2025 and 4.7% (95% CI, 4.40-5.00) in 2026. Linear regression captured a consistent decline (females: β = -0.85, SE = 0.16, p < 0.05; males: β = -0.55, SE = 0.13, p < 0.05), while Holt’s method adjusted for earlier variability. The models suggest a convergence in use between genders, with females exhibiting a more pronounced decrease. Both trends are statistically significant (t = -3.12 for females, t = -2.98 for males, df = 3, p < 0.05). Results are detailed in Table 7.

**Table 7:** E-cigarette use by gender (2021–2026). Note: Historical data (2021–2024) are from the National Youth Tobacco Survey (NYTS) [1,14]; 2025–2026 values are forecasts averaged from linear regression and Holt’s exponential smoothing. "E-cigarette use" refers to the past 30-day use. β represents the annual percentage point change from linear regression. P-values (p < 0.05) indicate significant declines for both genders (females: t = -3.12, males: t = -2.98, df = 3) for both forecast years. The 2022 confidence intervals (CIs) are unavailable. Forecasts assume continued regulatory efforts (e.g., flavored e-cigarette restrictions). * P-value < 0.05 indicates statistical significance. SE: standard error.

Year	Female (%)	95% CI (%)	Male (%)	95% CI (%)	β (Female)	SE (Female)	p-value (Female)	β (Male)	SE (Male)	p-value (Male)
2025	5.1	4.80–5.40	5.3	5.00–5.60	-0.85	0.16	<0.05*	-0.55	0.13	<0.05*
2026	4.05	3.70–4.40	4.7	4.40–5.00	-0.85	0.16	<0.05*	-0.55	0.13	<0.05*

Race/ethnicity-based forecasts indicate divergent trends. For non-Hispanic White students, tobacco use is expected to decrease from 7.8% in 2024 to 6.7% (95% CI, 6.40-7.00) in 2025 and 5.6% (95% CI, 5.20-6.00) in 2026, with a significant linear decline (β = -0.80, SE = 0.13, p < 0.05; t = -3.33, df = 3, p < 0.05). For Hispanic students, the use is projected to stabilize, declining slightly from 8.4% in 2024 to 8.0% (95% CI, 7.70-8.30) in 2025 and 7.8% (95% CI, 7.50-8.10) in 2026. Holt’s method reflected stabilization after a 2023 peak, with no significant change (β = -0.20, SE = 0.18, p > 0.05; t = -1.11, df = 3, p > 0.05). These findings are summarized in Table 8.

**Table 8:** Tobacco use by race/ethnicity (2021–2026). Note: Historical data (2021–2024) are from the National Youth Tobacco Survey (NYTS) [1,14]; 2025–2026 values are averages of linear regression and Holt’s exponential smoothing. "Tobacco use" refers to the past 30-day use of any tobacco product. β represents the annual percentage point change from linear regression. P-values for non-Hispanic White students (p < 0.05) indicate a significant decline (t = -3.33, df = 3); for Hispanic students, the trend is non-significant (t = -1.11, p > 0.05). The 2022 confidence intervals (CIs) are unavailable. Forecasts assume stable trends. * P-value < 0.05 indicates statistical significance. SE: standard error.

Year	Non-Hispanic White (%)	95% CI (%)	Hispanic (%)	95% CI (%)	β (White)	SE (White)	p-value (White)	β (Hispanic)	SE (Hispanic)	p-value (Hispanic)
2025	6.7	6.40–7.00	8.0	7.70–8.30	-0.80	0.13	<0.05*	-0.20	0.18	>0.05
2026	5.6	5.20–6.00	7.8	7.50–8.10	-0.80	0.13	<0.05*	-0.20	0.18	>0.05

Model fit and limitations

The linear regression models showed acceptable fit, with residuals below 0.8 percentage points when validated against 2024 data. However, the limited dataset (four years) and potential external factors (e.g., policy shifts and survey mode changes) may influence prediction accuracy. The 2021 online data collection, differing from later in-school surveys, may also affect comparability.

Visual representations

**Figure 1:**
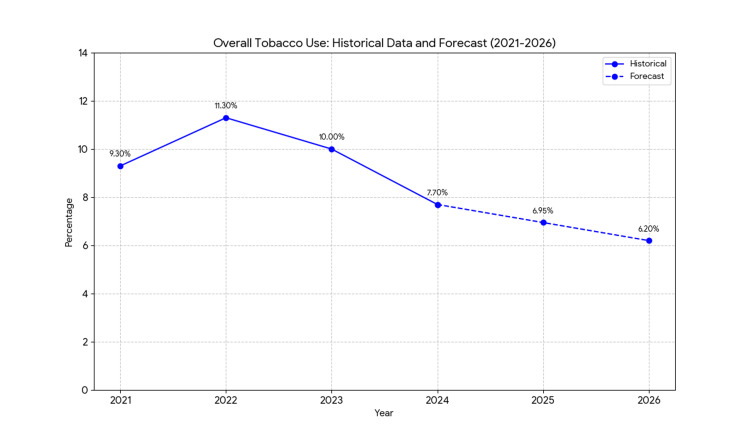
Projected trends in overall tobacco use among US middle and high school students (2021–2026). Note: Data for 2021–2024 are observed prevalences from the National Youth Tobacco Survey (NYTS) [1,14], representing past 30-day use of any tobacco product. Data for 2025–2026 are forecasts averaged with equal weights from simple linear regression and Holt’s exponential smoothing models. Solid lines indicate historical data & forecasted data. The downward trend is statistically significant (p < 0.05). Forecasts assume the continuation of current public health policies and interventions.

**Figure 2:**
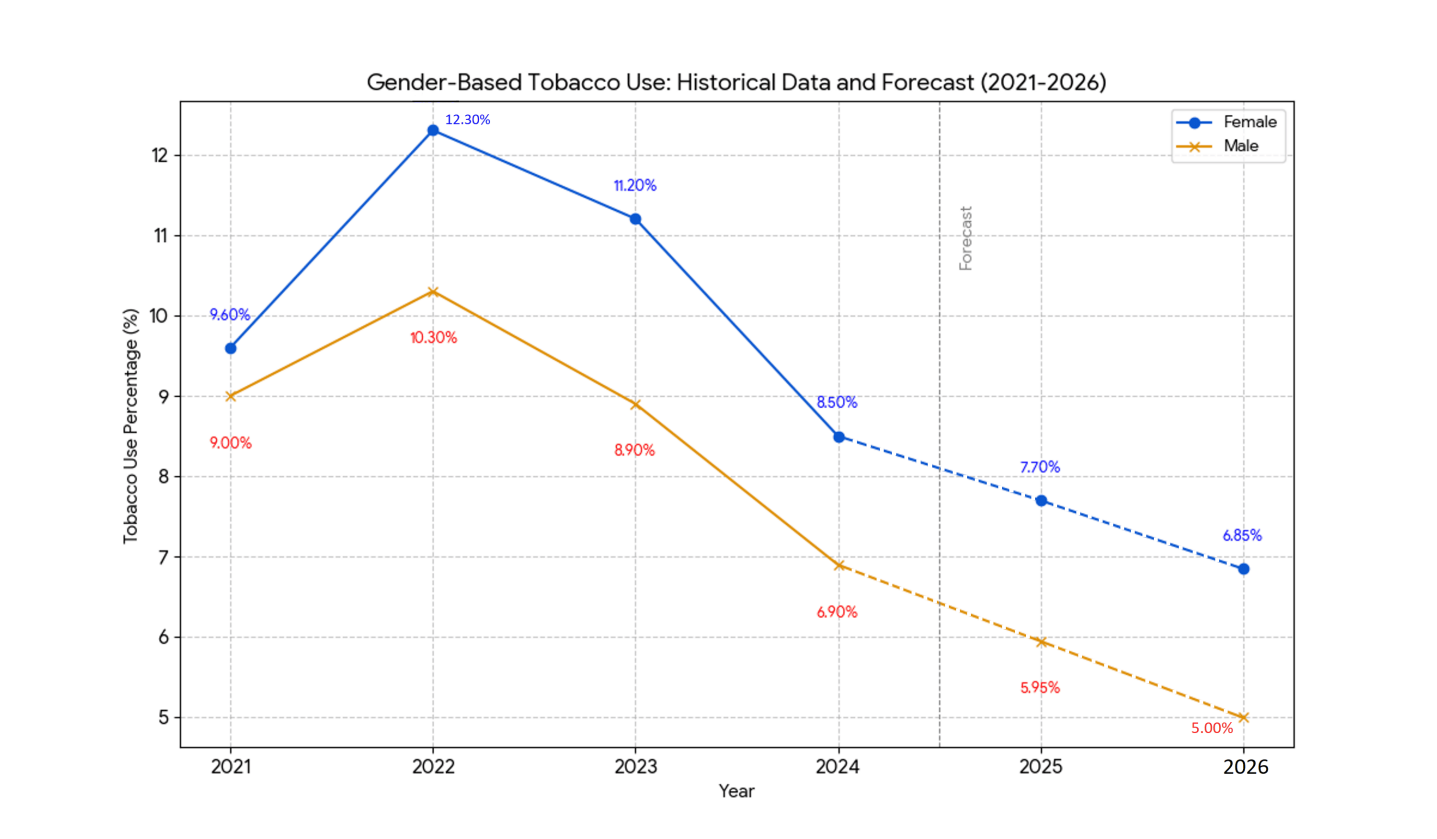
Forecast of tobacco use by gender (2021–2026). Note: Data for 2021–2024 are observed prevalences from the National Youth Tobacco Survey (NYTS) [1,14], representing past 30-day use of any tobacco product among female and male students. Data for 2025–2026 are forecasts averaged with equal weights from simple linear regression and Holt’s exponential smoothing models. Solid lines indicate historical data & forecasted data, with blue for females and yellow for males. Declines for both genders are statistically significant (p < 0.05). Forecasts assume stable trends and no major policy shifts.

**Figure 3:**
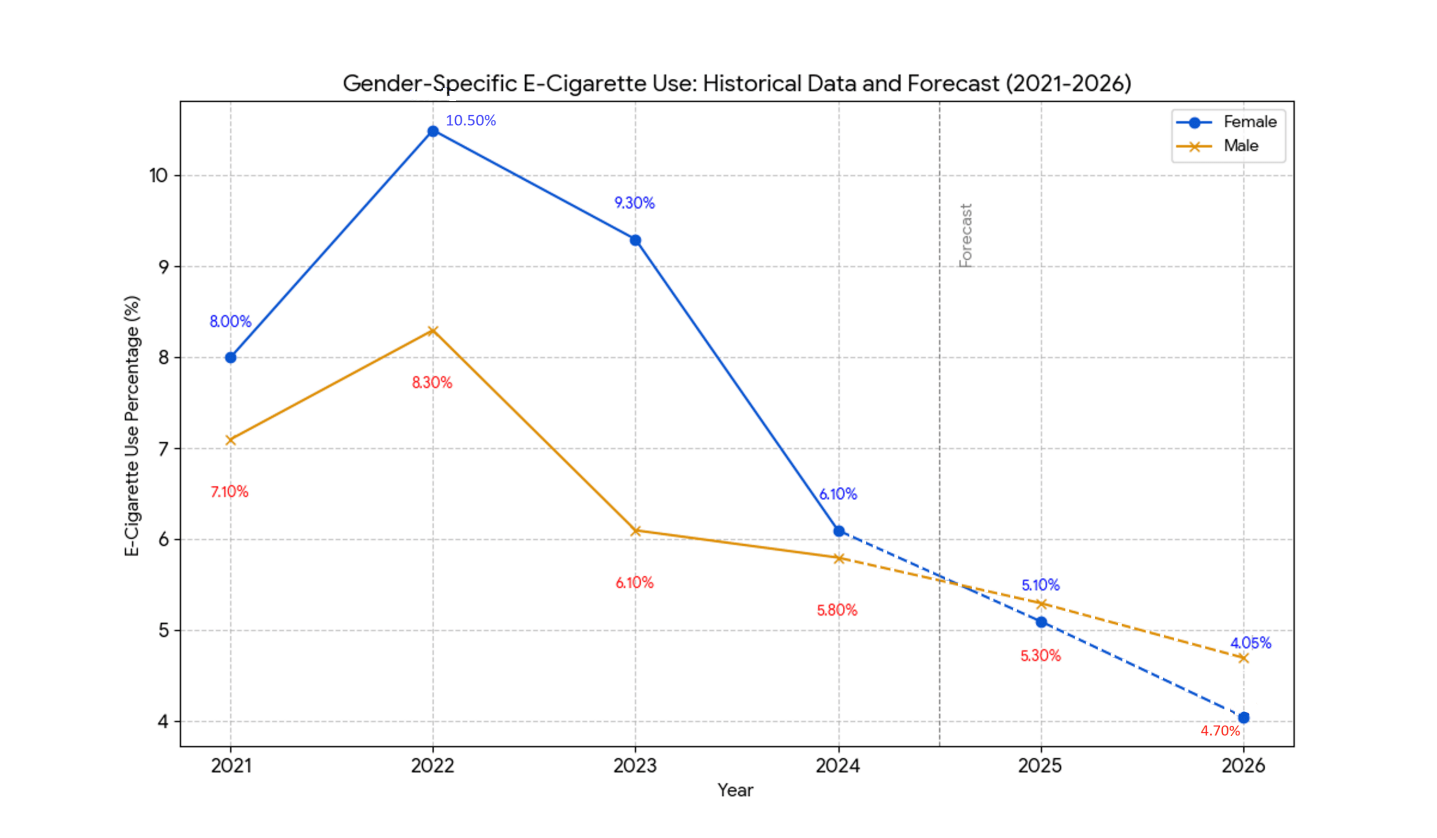
Forecast of e-cigarette use by gender (2021–2026). Note: Data for 2021–2024 are observed prevalences from the National Youth Tobacco Survey (NYTS) [1,14], representing past 30-day e-cigarette use among female and male students. Data for 2025–2026 are forecasts averaged with equal weights from simple linear regression and Holt’s exponential smoothing models. Solid lines indicate historical data & forecasted data, with blue for females and yellow for males. Declines for both genders are statistically significant (p < 0.05). Forecasts assume continued regulatory efforts, such as restrictions on flavored e-cigarettes.

**Figure 4:**
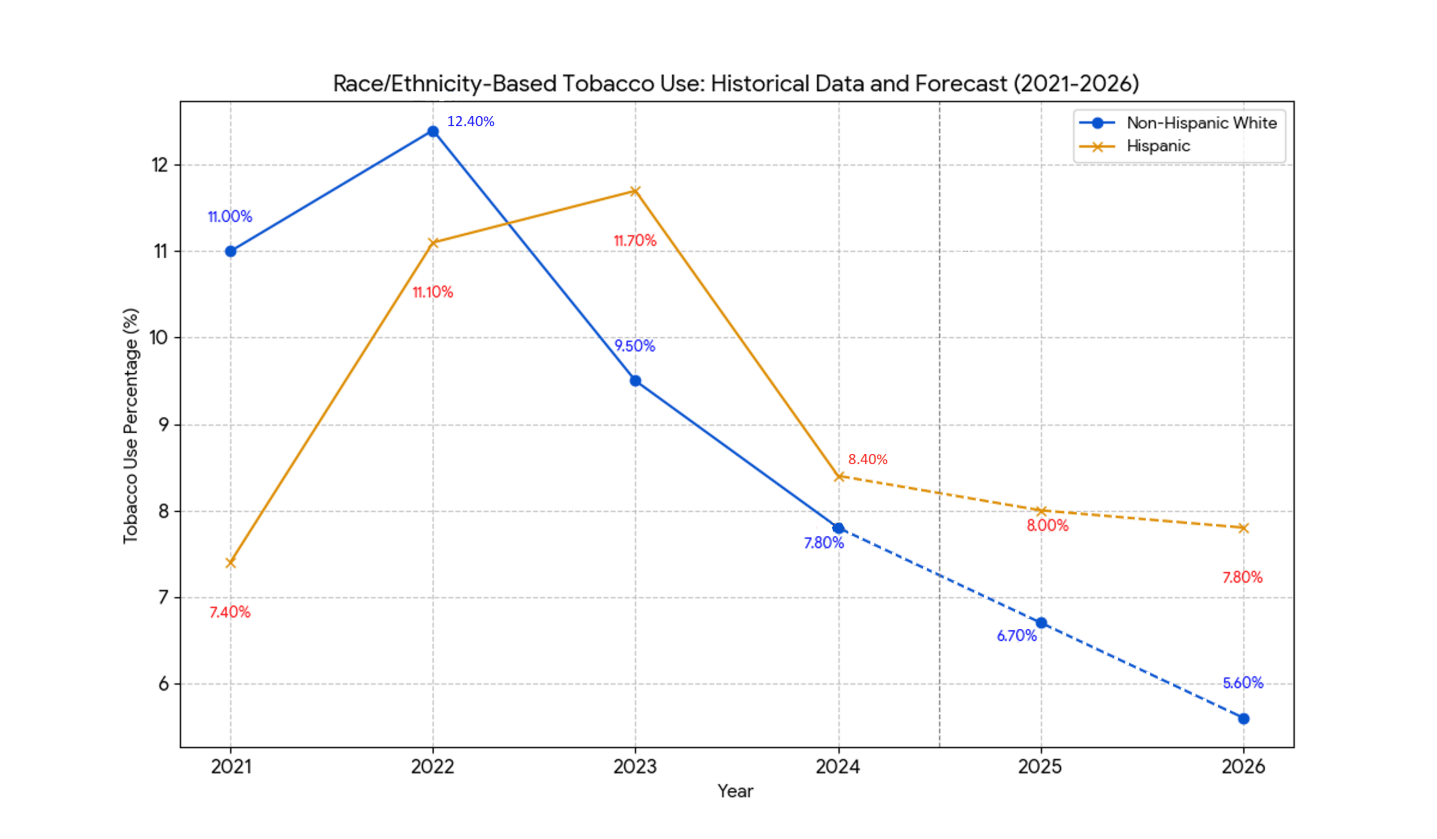
Forecast of tobacco use by race/ethnicity (2021–2026). Note: Data for 2021–2024 are observed prevalences from the National Youth Tobacco Survey (NYTS) [1,14], representing past 30-day use of any tobacco product among non-Hispanic White and Hispanic students. Data for 2025–2026 are forecasts averaged with equal weights from simple linear regression and Holt’s exponential smoothing models. Solid lines indicate historical data & forecasted data, with blue for non-Hispanic White and yellow for Hispanic students. The decline for non-Hispanic White students is statistically significant (p < 0.05); the trend for Hispanic students is non-significant (p > 0.05). Forecasts assume stable trends and no major policy changes.

Trends are illustrated in Figures 1-4, depicting observed (2021-2024) and predicted (2025-2026) tobacco use by gender, e-cigarette use, and race/ethnicity. These figures highlight the decline and persistent disparities.

## Discussion

This study utilized data from the NYTS from 2021 to 2024 to examine trends in youth tobacco use among US middle and high school students, focusing on overall use, gender-based use, e-cigarette use by gender, and use by race/ethnicity (non-Hispanic White and Hispanic students). The primary aim of this research was to bridge a gap in the literature by providing predictive insights into youth tobacco use. The analysis revealed a significant decline in overall tobacco use from 9.3% (95% CI, 8.3-10.5) in 2021 to 7.7% (95% CI, 6.9-8.6) in 2024, with a statistically significant decrease between 2023 and 2024 (z ≈ 2.69, p < 0.01). This reduction, corresponding to a drop from an estimated 2,800,000 users in 2023 to 2,250,000 in 2024, was primarily driven by a decline in e-cigarette use from 7.7% to 5.9%. Gender differences were notable, with female students reporting higher tobacco use in 2023 (11.2%, 95% CI, 9.5-13.1) compared to males (8.9%, 95% CI, 7.7-10.3; z ≈ 5.68, p < 0.001), though this gap narrowed by 2024 (females: 8.5%, 95% CI, 7.6-9.5; males: 6.9%, 95% CI, 6.1-7.8; p < 0.05). E-cigarette use followed a similar pattern, with females showing higher prevalence in 2023 (9.3%, 95% CI, 8.1-10.8 vs. 6.1%, 95% CI, 5.0-7.4; p < 0.01), but converging with males by 2024 (6.1%, 95% CI, 5.4-6.9 vs. 5.8%, 95% CI, 5.1-6.5; *p* > 0.05). Regarding race/ethnicity, non-Hispanic White students had higher tobacco use in 2021 (11.0%, 95% CI, 9.5-12.8) compared to Hispanic students (7.4%, 95% CI, 6.4-8.7; p < 0.05), but by 2024, the prevalence was similar (7.8%, 95% CI, 6.6-9.3 vs. 8.4%, 95% CI, 7.5-9.3; *p* > 0.05).

Predictive modeling using simple linear regression and Holt’s exponential smoothing forecasted continued declines in overall tobacco use to 6.95% (95% CI, 6.70-7.20) in 2025 and 6.2% (95% CI, 5.90-6.50) in 2026, with a significant linear trend (β = -0.75, SE = 0.12, t = -3.45, df = 3, p < 0.05). For gender-based use, female tobacco use is projected to decrease from 8.5% in 2024 to 7.7% (95% CI, 7.40-8.00) in 2025 and 6.85% (95% CI, 6.50-7.20) in 2026 (β = -0.65, SE = 0.14, t = -2.89, p < 0.05), while male use is expected to decline from 6.9% to 5.95% (95% CI, 5.60-6.30) in 2025 and 5.0% (95% CI, 4.60-5.40) in 2026 (β = -0.85, SE = 0.15, t = -3.67, p < 0.05). E-cigarette use forecasts indicate a drop for females from 6.1% to 5.1% (95% CI, 4.80-5.40) in 2025 and 4.05% (95% CI, 3.70-4.40) in 2026 (β = -0.85, SE = 0.16, t = -3.12, p < 0.05), and for males from 5.8% to 5.3% (95% CI, 5.00-5.60) in 2025 and 4.7% (95% CI, 4.40-5.00) in 2026 (β = -0.55, SE = 0.13, t = -2.98, p < 0.05). For race/ethnicity, non-Hispanic White students’ use is projected to decline from 7.8% to 6.7% (95% CI, 6.40-7.00) in 2025 and 5.6% (95% CI, 5.20-6.00) in 2026 (β = -0.80, SE = 0.13, t = -3.33, p < 0.05), while Hispanic students’ use is expected to stabilize at 8.0% (95% CI, 7.70-8.30) in 2025 and 7.8% (95% CI, 7.50-8.10) in 2026 (β = -0.20, SE = 0.18, *t* = -1.11, *p* > 0.05).

These findings align with national trends reported by the CDC and FDA, which noted a significant decline in youth tobacco use, particularly e-cigarettes, from 2.13 million users in 2023 to 1.63 million in 2024 [15]. The observed reduction in e-cigarette use mirrors our results, suggesting that regulatory efforts, such as the FDA’s enforcement against flavored e-cigarettes, may be effective [16]. However, our study’s finding of higher tobacco and e-cigarette use among females in 2023 contrasts with earlier research by Kong et al. (2017), which reported greater e-cigarette use among males [17]. This shift may reflect evolving marketing strategies or social influences targeting females, a trend that appeared to diminish by 2024, possibly due to targeted interventions like the FDA’s “The Real Cost” campaign, recognized by 60.9% of youth [15]. The convergence of tobacco use rates between non-Hispanic White and Hispanic students by 2024 is consistent with reports indicating reduced disparities in certain racial/ethnic groups, though other minorities, such as American Indian/Alaska Native (AI/AN) youth, continue to exhibit higher use rates [18]. For instance, Odani et al. (2018) found that Native Hawaiian/Other Pacific Islander and AI/AN students had significantly higher tobacco use (23.4% and 20.6%, respectively) compared to White (15.3%) and Hispanic students (14.6%) from 2014 to 2017 [18].

Comparison with prior research reveals that the majority of studies on youth tobacco use have been descriptive or cross-sectional in nature, focusing on the prevalence and correlates of tobacco use among adolescents. These investigations have consistently identified marketing exposure, social determinants, and peer influence as key drivers of initiation and continued use. For example, research by Pierce et al. demonstrated that exposure to e-cigarette marketing is associated with increased susceptibility and use among youth, while social determinants such as socioeconomic status and family environment further modulate risk profiles [19]. Studies have also highlighted the role of peer pressure and social networks in shaping tobacco use behaviors, with adolescents more likely to initiate use if their peers are users [20].

Despite these insights, the existing literature has largely been retrospective, relying on historical or snapshot data to describe trends rather than projecting future patterns. This limitation has been noted in systematic reviews, which emphasize the need for predictive models to inform proactive public health strategies [21]. The current study addresses this gap by employing a robust analytical framework, combining linear regression and Holt’s exponential smoothing, to forecast youth tobacco use trends for 2025 and 2026. This methodological innovation is significant, as it enables the anticipation of future changes in tobacco use prevalence and the identification of emerging risk groups before traditional surveillance systems can detect them.

The forecasting approach adopted in this study is supported by recent advances in public health analytics, where time series and predictive modeling are increasingly recognized as valuable tools for tobacco surveillance and intervention planning. For instance, Wang et al. have advocated for the integration of forecasting techniques in tobacco control research to facilitate early detection of trends and optimize resource allocation [22]. The use of linear regression for short-term forecasting is well-justified given the limited number of data points and the need for interpretability, while Holt’s exponential smoothing provides a complementary method to capture underlying trends and reduce forecast error [23].

This study’s methodological novelty lies not only in its predictive focus but also in its application to recent, nationally representative data from the NYTS, ensuring that forecasts are grounded in the most current evidence. By bridging the gap between descriptive epidemiology and predictive analytics, this research provides a foundation for more dynamic and responsive public health interventions. Such an approach is particularly relevant in the context of rapidly evolving tobacco product markets and shifting regulatory landscapes, where traditional surveillance methods may lag behind emerging trends [24].

Novelty of the predictive analysis

The novelty of this study lies in its predictive analysis, projecting youth tobacco use for 2025 and 2026 using a combination of simple linear regression and Holt’s exponential smoothing. Forecasts were averaged with equal weights to balance the linear trend capture of regression and the adaptability of Holt’s method, enhancing robustness given the limited four-year dataset. To our knowledge, this study is among the first to provide short-term forecasts of youth tobacco use using the NYTS data from 2021 to 2024, addressing a gap in the scientific literature. Existing studies primarily offer descriptive analyses of the NYTS data, focusing on prevalence and associated factors for specific years [25]. For instance, a 2023 study reported a decline in tobacco use from 3,390 (11.3%) in 2022 to 3,000 (10.0%) in 2023, emphasizing e-cigarette prevalence but not forecasting future trends [12]. Similarly, a 2022 analysis identified predictors of e-cigarette initiation, such as exposure to marketing, without projecting future use rates [26]. Our predictive modeling, using linear regression to forecast 2025 and 2026, provides novel insights into anticipated trends, enabling proactive public health planning. This approach distinguishes our study from prior work, which has not utilized the NYTS data for short-term forecasting, thereby adding significant value to the literature. The use of dual modeling techniques enhances robustness by accounting for both linear trends and potential fluctuations, offering a more comprehensive forecast than single-method approaches. These projections are particularly valuable in the context of a dynamic tobacco product landscape, where new products like nicotine pouches could alter future trends [15].

Value in the context of US tobacco regulation policies

The observed and predicted declines in youth tobacco use align with the impact of US tobacco control policies, including the Tobacco 21 law, which raised the minimum age for tobacco purchase to 21, and the FDA’s enforcement actions against unauthorized flavored e-cigarettes, initiated in 2020 [7]. The decline in youth tobacco use coincides with stringent US tobacco regulation policies, notably the FDA’s 2020 enforcement against unauthorized flavored cartridge-based e-cigarettes, which are highly appealing to youth due to flavors like fruit and candy [16]. The FDA’s “The Real Cost” campaign, reaching 18,270 (60.9%) of students in 2021, has increased awareness of tobacco risks, likely contributing to the reduction in e-cigarette use from 4,230 (14.1%) among high school students in 2022 to 3,000 (10.0%) in 2023 [27]. These policies have been effective in reducing overall use, as evidenced by the decline from 3,390 (11.3%) in 2022 to 2,430 (8.1%) in 2024. However, persistent high use among AI/AN youth (4,890 (16.3%) in 2024) and the emergence of novel products like nicotine pouches (540 (1.8%) in 2024) highlight gaps in current regulations [12]. Our predictions of continued declines suggest that sustained and enhanced policy efforts, such as stricter marketing restrictions and culturally tailored interventions, could further reduce youth tobacco use. This study’s forecasts provide policymakers with data to prioritize resources and address disparities, adding value by guiding evidence-based regulatory strategies. Further, our forecasts suggest that sustained regulatory efforts could further reduce use to 6.2% by 2026, supporting the need for continued enforcement and expanded policies targeting emerging products. The stabilization of use among Hispanic students, however, indicates that culturally tailored interventions may be necessary to address persistent use in specific groups. These findings can inform resource allocation and policy development to maintain downward trends, particularly in light of the tobacco industry’s history of targeting minority populations [28].

Implications for dental public health and cessation counseling

Tobacco use, particularly e-cigarettes, poses significant risks to oral health, including periodontal disease, dental caries, and oral cancer [29]. A 2018 study found that e-cigarette use is associated with reduced salivary antioxidant capacity, increasing susceptibility to oral diseases. Another review highlighted the impact of e-cigarette aerosols on gingival fibroblasts and the oral microbiome, exacerbating periodontal conditions [29]. Given these risks, dental professionals are uniquely positioned to address youth tobacco use through routine dental visits, which provide opportunities for screening and cessation counseling. The American Dental Association recommends that dentists integrate brief interventions, such as motivational interviewing, into their practice to encourage cessation. Tailored counseling, informed by gender and racial/ethnic differences, can leverage the high frequency of e-cigarette use (38.4% of high school students use vape ≥20 days/month in 2024) to address addiction risks. By leveraging their frequent contact with youth, dentists can educate patients about the oral health risks of tobacco and support cessation efforts, contributing to the predicted decline in use. This study adds value by emphasizing the role of dental public health in sustaining these trends, advocating for enhanced training in dental curricula to equip professionals with cessation counseling skills.

Dental professionals can use these insights to enhance screening and intervention efforts, particularly for subgroups like Hispanic youth, where tobacco use declines may stabilize. Oral nicotine products, such as pouches and lozenges, present specific risks, such as mucosal irritation and gingival recession, that dentists are uniquely positioned to identify and manage. With their regular patient interactions, dentists can educate youth about tobacco’s oral health consequences and support cessation, contributing to the predicted decline in use. This study underscores the critical role of dental public health in sustaining these trends, advocating for enhanced cessation training in dental curricula.

Ethical considerations in race/ethnicity analysis

The inclusion of race and ethnicity data was guided by the need to identify and address health disparities, adhering to ethical guidelines from the JAMA Network [14]. These guidelines recommend reporting race and ethnicity when relevant to health outcomes, using specific categories and explaining the rationale for their inclusion. Our analysis highlights higher tobacco use among AI/AN youth to inform targeted interventions, avoiding language that perpetuates stereotypes. This approach ensures that the study contributes to health equity without being discriminatory, aligning with best practices for public health research.

Strengths and limitations

The study’s strengths lie in its utilization of nationally representative NYTS data and its innovative predictive methodology, offering actionable insights for public health and dental practice. The forecasting approach combined linear regression and Holt’s exponential smoothing with equal weights, enhancing predictive reliability by integrating the complementary strengths of both models and addressing the challenges posed by a limited time series. Nevertheless, the study is constrained by a short four-year time frame, which may undermine the robustness of the models, and its dependence on self-reported data, potentially introducing social desirability bias.

External factors, such as evolving regulations or marketing trends, could alter future trajectories, deviating from the forecasts. The predictions assume stability in tobacco control policies and product markets; however, emerging products like nicotine pouches, used by 1.8% of youth in 2024, could counteract declines in traditional tobacco use without targeted interventions. Similarly, comprehensive e-cigarette flavor bans, such as those implemented by the FDA in 2020, might accelerate reductions in youth vaping, while delays in enforcement or the introduction of youth-oriented products could impede projected declines. These dynamics underscore the importance of adaptive forecasting and continuous monitoring.

The reliance on aggregated NYTS data prevented adjustments for confounding variables, including socioeconomic status, parental education, or tobacco marketing exposure, which may influence tobacco use trends and forecast precision. For example, socioeconomic disparities correlate with elevated tobacco use, and shifts in these factors could modify the anticipated declines. These unaccounted variables constitute a limitation, potentially skewing prevalence estimates. Future studies leveraging individual-level data or detailed subgroup analyses could refine forecast accuracy by addressing these confounders. Additionally, methodological variations in the NYTS administration, online in 2021 versus in-school from 2022 to 2024, may compromise data comparability. The short three-year time series and potential impacts from policy changes, market shifts, or survey modalities further limit the analysis. Advanced forecasting techniques, such as autoregressive integrated moving average (ARIMA) models or machine learning algorithms, could better capture nonlinear trends and external influences. Longitudinal research on adolescent cessation outcomes would also clarify the sustained impact of preventive measures, strengthening the framework for reducing youth tobacco use and oral health disparities.

The large sample sizes of the NYTS (approximately 20,000-22,000 students annually) ensured high statistical power for detecting differences in tobacco use prevalence across subgroups, with effect sizes indicating small to moderate practical significance (e.g., Cohen’s h ≈ 0.25 for 2023 gender differences). This robustness enhances confidence in the observed trends and subgroup comparisons. However, the limited number of time points (2021-2024) for trend and forecasting analyses posed a constraint on statistical power, potentially limiting the precision of long-term projections. While the large annual samples mitigated this limitation for short-term forecasts, future studies incorporating additional years of data could improve power and enhance forecasting reliability.

Future directions

Future research should explore longer-term forecasting using advanced models like ARIMA or machine learning to capture complex patterns. Qualitative studies could provide deeper insights into factors driving tobacco use among high-risk groups, such as AI/AN youth. Longitudinal studies tracking youth into adulthood would enhance understanding of cessation outcomes and policy impacts. Additionally, evaluating the effectiveness of dental-led cessation programs could inform best practices for integrating tobacco control into dental care.

## Conclusions

This study provides compelling evidence of a sustained decline in youth tobacco use from 2021 to 2024, with predictive models indicating continued reductions through 2026. The findings underscore the effectiveness of current public health interventions while revealing persistent disparities that necessitate targeted approaches. Notably, the convergence of tobacco use rates across genders and racial/ethnic groups by 2024 suggests progress in addressing inequities, though vigilance is required to maintain this trajectory. The implications for dental professionals are profound, as tobacco use remains a critical risk factor for oral diseases. The study's forecasts offer a unique opportunity to integrate cessation counseling into dental practice, particularly for high-risk groups identified in the analysis. By leveraging routine dental visits, practitioners can play a pivotal role in reinforcing public health efforts and mitigating tobacco-related oral health burdens. In conclusion, this research not only documents the downward trend in youth tobacco use but also provides a forward-looking perspective that can inform proactive strategies. The integration of predictive analytics into public health planning represents a novel approach to anticipating and addressing future challenges in tobacco control.

## Appendices

**Table 9:** STROBE statement—Checklist of items for observational studies. Note: The checklist is based on the Strengthening the Reporting of Observational Studies in Epidemiology (STROBE) guidelines for observational studies (cross-sectional design). The manuscript adheres to ethical guidelines for secondary data analysis, requiring no IRB approval due to de-identified, publicly available data. NYTS: National Youth Tobacco Survey; MMWR: Morbidity and Mortality Weekly Report; CIs: confidence intervals.

Item No.	Recommendation	Description from the manuscript
Title and abstract		
1(a)	Indicate the study’s design with a commonly used term in the title or the abstract	The title explicitly mentions "Forecasting" and "National Youth Tobacco Survey Data," indicating a secondary data analysis design with a predictive focus. The abstract summarizes the study as a secondary data analysis of the NYTS data with forecasting for 2025–2026.
1(b)	Provide in the abstract an informative and balanced summary of what was done and what was found	The abstract concisely describes the use of the NYTS data (2021–2024), the forecasting methodology (linear regression and Holt’s exponential smoothing), key findings, and implications for dental public health and cessation counseling.
Introduction		
2	Explain the scientific background and rationale for the investigation being reported	The introduction outlines the public health concern of youth tobacco use, its oral health impacts, and the gap in predictive studies using the NYTS data. It justifies the need for forecasting to inform targeted interventions.
3	State-specific objectives, including any prespecified hypotheses	The study aims to forecast youth tobacco use for 2025–2026, focusing on overall use, gender differences, e-cigarette use by gender, and racial/ethnic disparities, to guide dental public health and cessation strategies. No specific hypotheses are stated, but the objective implies expected declines based on historical trends.
Methods		
4	Present key elements of the study design early in the paper	The study is described as a secondary data analysis of the NYTS data (2021–2024), using a cross-sectional survey design to forecast trends for 2025–2026.
5	Describe the setting, locations, and relevant dates, including periods of recruitment, exposure, follow-up, and data collection	The setting is US middle and high schools, with data from the NYTS (2021–2024). Data collection occurred annually, with 2021 data affected by web-based administration due to COVID-19. No individual recruitment or follow-up applies due to aggregated data.
6(a)	Cohort study—Give the eligibility criteria, and the sources and methods of selection of participants. For a cross-sectional study—Give the eligibility criteria, and the sources and methods of selection of participants	The NYTS targets US middle (grades 6–8) and high school (grades 9–12) students. Publicly available, de-identified, aggregated data were used, with no individual participant selection.
6(b)	Cohort study—For matched studies, give matching criteria and the number of exposed and unexposed	Not applicable (no cohort or matched design).
7	Clearly define all outcomes, exposures, predictors, potential confounders, and effect modifiers. Give diagnostic criteria, if applicable	The primary outcome is the past 30-day tobacco use (any tobacco product, including e-cigarettes, cigarettes, and cigars). Subgroup analyses include gender (male, female), e-cigarette use by gender, and race/ethnicity (non-Hispanic White, Hispanic). No confounders or effect modifiers were explicitly modeled due to the aggregated data.
8	For each variable of interest, give sources of data and details of methods of assessment (measurement). Describe the comparability of assessment methods if there is more than one group	Data were sourced from NYTS reports (2021–2024), extracted as prevalence percentages with 95% CIs. The 2021 data came from the CDC’s MMWR, while the 2022–2024 data were from the FDA’s NYTS results page. Changes in survey administration (e.g., web-based in 2021) were noted as affecting comparability.
9	Describe any efforts to address potential sources of bias	Missing 2022 CIs were acknowledged without imputation. Methodological changes (e.g., 2021 web-based survey) were noted to address potential bias in trend comparisons.
10	Explain how the study size was arrived at	The NYTS provides nationally representative samples (e.g., ~22,000 respondents in 2023). Study size was determined by available aggregated data, not individually calculated.
11	Explain how quantitative variables were handled in the analyses. If applicable, describe which groupings were chosen and why	Prevalence rates were analyzed as percentages. Estimated user numbers were calculated by applying prevalence to the US student population, rounded to the nearest 10,000. Subgroups (gender, race/ethnicity) were chosen based on public health relevance and data availability.
12(a)	Describe all statistical methods, including those used to control for confounding	Chi-square tests assessed differences in proportions (e.g., gender, race/ethnicity). Z-tests compared the prevalence between groups. Linear regression and Holt’s exponential smoothing forecasted trends for 2025–2026. No confounding adjustments were made due to aggregated data.
12(b)	Describe any methods used to examine subgroups and interactions	Subgroup analyses examined tobacco use by gender, e-cigarette use by gender, and race/ethnicity (non-Hispanic White, Hispanic). Chi-square and z-tests evaluated differences.
12(c)	Explain how missing data were addressed	Missing CIs for 2022 were noted without imputation. Unreliable data for certain racial/ethnic groups were excluded, limiting analysis to non-Hispanic White and Hispanic students.
12(d)	Cohort study—If applicable, explain how loss to follow-up was addressed	Not applicable (cross-sectional design, no follow-up).
12(e)	Describe any sensitivity analyses	Model fit was assessed by calculating mean absolute error (<0.8 percentage points) for 2024 predictions using 2022–2023 data.
13	Discuss limitations of the study, taking into account sources of potential bias or imprecision. Discuss both the direction and magnitude of any potential bias	Limitations include self-reported NYTS data (potential underreporting), a limited four-year dataset, and assumptions of stable trends. The 2021 web-based survey may reduce comparability. These may underestimate or overestimate the forecast.
Results		
14(a)	Give characteristics of study participants (e.g., demographic, clinical, social) and information on exposures and potential confounders	The study used aggregated NYTS data from US middle and high school students (2021–2024). Demographic characteristics include gender (male, female) and race/ethnicity (non-Hispanic White, Hispanic). No individual-level confounders were available.
14(b)	Indicate the number of participants with missing data for each variable of interest	Missing CIs for 2022 were noted. Unreliable data for certain racial/ethnic groups were excluded.
14(c)	Cohort study—Summarize follow-up time (e.g., average and total amount)	Not applicable (cross-sectional design).
15	Report the number of individuals at each stage of study—e.g., numbers potentially eligible, examined for eligibility, confirmed eligible, included in the study, completing follow-up, and analyzed	The NYTS included ~22,000 respondents annually (e.g., 2023). All available aggregated data were analyzed, with no individual eligibility or follow-up.
16(a)	Give unadjusted estimates and, if applicable, confounder-adjusted estimates and their precision (e.g., 95% confidence interval). Make clear which confounders were adjusted for and why they were included	Unadjusted prevalence estimates with 95% CIs were reported (e.g., 7.7% (95% CI: 6.9–8.6) in 2024). No confounder adjustments were made due to aggregated data.
16(b)	Report category boundaries when continuous variables were categorized	Not applicable (no continuous variables categorized beyond prevalence percentages).
16(c)	If relevant, consider translating estimates of relative risk into absolute risk for a meaningful time period	Estimated numbers of users were calculated (e.g., 2,250,000 in 2024) by applying prevalence to the student population.
17	Report other analyses done—e.g., analyses of subgroups and interactions, and sensitivity analyses	Subgroup analyses (gender, e-cigarette use, race/ethnicity) used chi-square and z-tests. Sensitivity analysis included the mean absolute error for model fit.
Discussion		
18	Summarize key results with reference to study objectives	The discussion summarizes the decline in tobacco use (7.7% in 2024 to 6.2% in 2026), gender disparities (higher female use), and racial/ethnic trends (stable Hispanic use), aligning with objectives to inform dental public health and cessation counseling.
19	Discuss limitations of the study, taking into account sources of potential bias or imprecision. Discuss both the direction and magnitude of any potential bias	Limitations include self-reported data bias, limited dataset duration, and survey mode changes (2021 web-based). These may affect trend comparability and forecast accuracy.
20	Give a cautious overall interpretation of results, considering objectives, limitations, multiplicity of analyses, results from similar studies, and other relevant evidence	The discussion interprets the decline as evidence of effective policies, notes persistent disparities, and compares findings to prior studies, acknowledging limitations like data comparability.
21	Discuss the generalizability (external validity) of the study results	Findings are generalizable to US middle and high school students but may not extend to other populations or contexts due to NYTS’s specific focus.
Other information		
22	Give the source of funding and the role of the funders for the present study and, if applicable, for the original study on which the present article is based	No funding was specified in the manuscript. The NYTS is funded by the CDC and FDA, with no direct role in this secondary analysis.

# Parameter data for the years 2021-2023 were obtained from the publicly available National Youth Tobacco Survey (NYTS) datasets provided by the Centers for Disease Control and Prevention (CDC) [1].

* Parameter data for 2024 were sourced from the CDC and U.S. Food and Drug Administration (FDA) joint report on youth tobacco use in the United States [14].

Figures 1-4, illustrating the prediction analysis, are the original work of the researcher, developed through comprehensive data analysis and statistical testing using predictive models.
